# Loss of a Candidate Biliary Atresia Susceptibility Gene, *add3a,* Causes Biliary Developmental Defects in Zebrafish

**DOI:** 10.1097/MPG.0000000000001375

**Published:** 2016-10-24

**Authors:** Vivian Tang, Zenobia C. Cofer, Shuang Cui, Valerie Sapp, Kathleen M. Loomes, Randolph P. Matthews

**Affiliations:** ∗ Division of Gastroenterology, Hepatology, and Nutrition, The Children's Hospital of Philadelphia Research Institute; † Department of Pediatrics, Perelman School of Medicine, University of Pennsylvania, Philadelphia.

**Keywords:** biliary atresia, biliary development, liver development

## Abstract

**Objectives:**

Biliary atresia (BA) is a progressive fibroinflammatory cholangiopathy affecting the bile ducts of neonates. Although BA is the leading indication for pediatric liver transplantation, the etiology remains elusive. *Adducin 3* (*ADD3*) and *X-prolyl aminopeptidase 1* (*XPNPEP1*) are 2 genes previously identified in genome-wide association studies as potential BA susceptibility genes. Using zebrafish, we investigated the importance of *ADD3* and *XPNPEP1* in functional studies.

**Methods:**

To determine whether loss of either gene leads to biliary defects, we performed morpholino antisense oligonucleotide (MO) knockdown studies targeting *add3a* and *xpnpep1* in zebrafish. Individuals were assessed for decreases in biliary function and the presence of biliary defects. Quantitative polymerase chain reaction was performed on pooled 5 days postfertilization larvae to assess variations in transcriptional expression of genes of interest.

**Results:**

Although both *xpnpep1* and *add3a* are expressed in the developing zebrafish liver, only knockdown of *add3a* produced intrahepatic defects and decreased biliary function. Similar results were observed in homozygous *add3a* mutants. MO-mediated knockdown of *add3a* also showed higher mRNA expression of hedgehog (Hh) targets. Inhibition of Hh signaling rescued biliary defects caused by *add3a* knockdown. Combined knockdown of *add3a* and *glypican-1* (*gpc1*), another mediator of Hh activity that is also a BA susceptibility gene, resulted in more severe biliary defects than knockdown of either alone.

**Conclusions:**

Our results support previous studies identifying *ADD3* as a putative genetic risk factor for BA susceptibility. Our results also provide evidence that *add3a* may be affecting the Hh pathway, an important factor in BA pathogenesis.

## Abstract 2

Supplemental Digital Content is available in the text

**What Is Known**

- The etiology of biliary atresia is unknown, but evidence suggests that genetic susceptibility may play a role.
- A genome-wide association study identified a biliary atresia susceptibility locus on chromosome 10q24.2, in close proximity to the *add3* and *xpnpep1* genes.

**What Is New**

- Loss of the zebrafish ortholog *add3a*, but not *xpnpep1*, leads to intrahepatic defects and impaired biliary function.
- *add3a* morphants show upregulation of hedgehog pathway target genes, and inhibition of hedgehog rescues the phenotype.
- Combined knockdown of *add3a* and *gpc1*, another known biliary atresia susceptibility gene, results in a more severe phenotype, indicating that these 2 genes may act within the same pathway.
- Functional studies of candidate biliary atresia susceptibility genes can shed light on possible mechanisms of injury and pathogenesis of disease and biliary development.

Biliary atresia (BA) is an idiopathic, progressive, fibroinflammatory cholangiopathy that occurs exclusively in neonates. The incidence of BA ranges from 1 per 10,000 in the United States to up to 3.7 per 10,000 in parts of Asia (1,2). Until the advent of the Kasai hepatoportoenterostomy, BA was universally fatal. BA, however, continues to be the leading indication for pediatric liver transplantation worldwide.

Although the etiology of BA is unknown, it appears to be at least partially related to genetic factors affecting biliary development, as it presents exclusively in neonates and infants (3). Although BA is unlikely to be strictly a genetic disease, several studies have identified specific genes associated with BA (4). Recently, investigators using genome-wide association studies (GWASs) reported significant association with the chromosomal region around 10q24.2 for BA susceptibility in Asian (5,6) and Caucasian-American (7) populations. This region overlaps with the *X-prolyl aminopeptidase 1* (*XPNPEP1*) and *gamma-adducin* (*ADD3*) genes (8). *XPNPEP1* is found in hepatobiliary epithelial cells and encodes an enzyme that promotes the degradation of bradykinin and substance P (7,9), bioactive peptides involved in inflammation (10). Substance P also has an anticholeretic effect on hepatic bile flow (11) and is elevated in the serum of patients with cholestatic liver disease (12). *ADD3* belongs to a family of actin cytoskeletal proteins involved in cell-cell contact in epithelial tissues (13). *ADD3* is also abundantly expressed in the biliary tract of the fetal liver (14). Thus, both *ADD3* and *XPNPEP1* appear to be reasonable candidates for mediating BA susceptibility, because they are expressed in the liver and both intrahepatic and extrahepatic bile ducts, and are important in pathways thought to be involved in BA pathogenesis and hepatobiliary development.

In the present study, we used zebrafish models to determine whether *add3a* or *xpnpep1*, the zebrafish homologs of human *ADD3* and *XPNPEP1*, has functional roles in biliary development. Although both *add3a* and *xpnpep1* are expressed in the developing zebrafish liver, we show that only a loss of *add3a* expression leads to decreased biliary function and biliary defects. We show that inhibition of hedgehog (Hh) signaling rescues biliary defects induced by *add3a* knockdown, suggesting that *add3a* may have a role in the Hh pathway. Interestingly, combined knockdown of *add3a* and *gpc1*, a gene we previously reported as involved in BA pathogenesis by upregulating Hh signaling (15), has an epistatic effect on biliary development. Our findings support and further the GWAS by demonstrating that *ADD3*, not *XPNPEP1*, is the most likely gene influencing BA susceptibility, and in addition we provide evidence suggesting a possible pathogenic mechanism.

## MATERIALS AND METHODS

### Zebrafish Lines

*Add3a* knockdown experiments were performed on wild-type Tupfel long fin zebrafish embryos, raised in the Children's Hospital of Philadelphia animal facility according to approved protocols. Zebrafish embryos carrying a mutation in *add3a,* designated sa819, were obtained from the Zebrafish International Resource Center (ZIRC) and raised and bred in the Children's Hospital of Philadelphia animal facility as mentioned above. The sa819 fish carry a point mutation in the *add3a* gene that leads to a nonsense mutation (ZIRC). Homozygous mutants were determined by the appearance of heart edema at 2 days post fertilization (2 dpf), and the presence of the T>A change in individuals bearing the phenotype was confirmed by Sanger sequencing. Polymerase chain reaction (PCR) primers used for Sanger sequencing are listed in Supplemental Digital Content, Table 1. All procedures involving zebrafish were conducted in accordance with federal guidelines and approved Institutional Animal Care and Use Committee protocols. All animals received humane care according to the criteria outlined in the “Guidelines for the Care and Use of Laboratory Animals.”

### In Situ Hybridization Studies

PCR primers (Supplemental Digital Content, Table 1) were used to design antisense riboprobes for zebrafish *add3* and *xpnpep* orthologs. Complementary DNA (cDNA) templates for *xpnpep1*, *xpnpep2*, *add1*, *add2*, *add3a*, and *add3b* were obtained using a T3 promoter sequence starting at the 5’ end of the reverse primer, as shown in the sequences in Supplemental Digital Content, Table 1. Treated larvae were sacrificed at 3 dpf then fixed in 4% paraformaldehyde. To remove pigmentation, fixed specimens were bleached in a solution containing 3% hydrogen peroxide and 1% potassium hydroxide, in accordance with standard protocols. In situ hybridization was performed on specimens as previously described (16).

### Morpholino Antisense Oligonucleotide Design and Injection

Morpholino antisense oligonucleotides (MO) designed to target either *add3a* or *xpnpep1* were obtained from GeneTools (Philomath, OR). Targeted sequences for blocking either a 5’ translational start site or a splice acceptor site are shown in Supplemental Digital Content, Table 1. Approximately 1.5 ng of morpholino was injected in embryos ranging from 1 to 8 cell stage and subsequently titrated to establish experimental dose. Quantitative PCR (qPCR) analysis on cDNA derived from morpholino-injected larvae from either *add3a* or *xpnpep1* was performed to confirm knockdown of spliced mRNA (Supplemental Digital Content, Fig. 1). Specificity of *add3a* MO−mediated knockdown was confirmed by rescuing the phenotype with injection of *add3a* mRNA (Supplemental Digital Content, Fig. 1) synthesized from a full-length *add3a* template construct (Addgene, Cambridge, MA) using mMessage mMachine (Ambion, Carlsbad, CA) as per the manufacturer's instructions.

For the *add3a* and *gpc1* epistatic studies, *add3a* and *gpc1* morpholinos were titrated so that individual low-dose concentrations did not produce significant impairment of biliary excretion, as assessed by gallbladder uptake of the quenched fluorescent phospholipid PED6. Morpholinos were combined so that individual low-dose concentrations were consistent with individual morpholino concentrations and injected into embryos between the 1–8 cell stage. Oligonucleotide sequences for *gpc1* appeared previously in Cui et al (15). For the Hh pathway inhibition studies, larvae were incubated in E3 containing cyclopamine at a final concentration of 10 μmol/L from 2 dpf to 5 dpf.

### PED6 Treatment

The quenched fluorescent phospholipid, PED6, was added to the water of 5 dpf embryos at a concentration of 0.1 μg/mL to allow direct visualization of the gallbladder (17). For morpholino-injected and mutant fish, PED6 uptake in the gallbladders was scored blindly as “normal,” “faint,” or “absent,” as in previous studies (18,19).

### Whole-Mount Immunofluorescence

To study biliary anatomy, 5 dpf larval livers were stained for cytokeratin, allowing visualization of intra- and extrahepatic bile ducts, as in several previous studies (20). Immunostained livers were examined using confocal microscopy, and images were processed using ImageJ and Adobe Photoshop, as previously described.

### Quantitative Polymerase Chain Reaction Studies of Gene Expression

qPCR was used to examine gene expression differences in the biliary transcription factor *vhnf1*, Notch target *her6*, Hh effector *gli2a*, and transforming growth factor beta 1β target connective tissue growth factor (*ctgf*). Other Hh targets that were used included *ptch1*, *foxl1*, *znf697*, and *ccnd1*. These primer sequences, including the normalizing primer pair for *hprt*, have been reported previously (15). For these studies, 5 dpf larvae were sacrificed and then stored in RNA later (Qiagen, Valencia, CA). Per standard protocols, RNA was isolated and reverse transcribed to generate cDNA (Ambion, Invitrogen). qPCR was performed as in our previous studies on an Applied Biosystems StepOne Plus qPCR machine and software (15) using Perfecta-SYBR-green Fastmix (Quanta Biosciences, Gaithersburg, MD).

### Statistical Tests

For comparison of the levels of PED6 uptake between groups, we used chi-square analysis similar to our previous studies (19). For the qPCR studies, Microsoft Excel was used to perform Student *t* test comparing groups, which were triplicate specimens, similar to previous studies (15).

## RESULTS

### Expression of *add3a* and *xpnpep1* in Developing Liver

**FIGURE 1:**
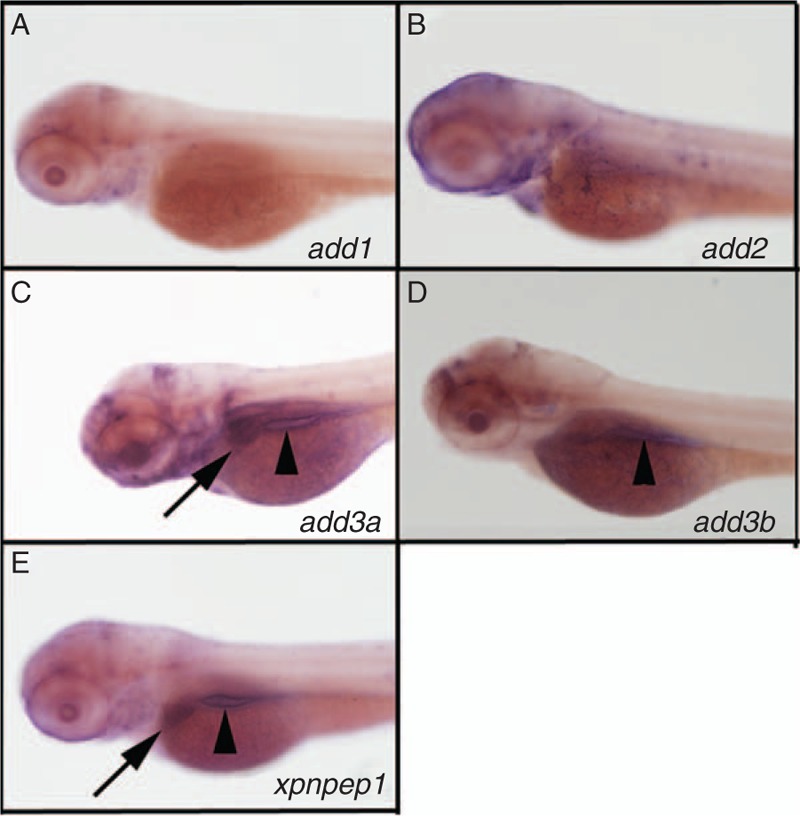
Expression of *add3a* and *xpnpep1* in the developing liver. Whole-mount in situ hybridization (ISH) of 3 days post fertilization (dpf) zebrafish larvae probed for *add1* (A), *add2* (B), *add3a* (C), *add3b* (D), and *xpnpep1* (E). There is liver staining for *add3a* (C, black arrow) and *xpnpep1* (E, black arrow) but not for the other probes. There is intestinal staining for *add3a*, *add3b* and *xpnpep1* (black arrowheads in C, D, E). There is also staining of *add2* and *add3a* in the head region, which is of unclear specificity (B, C). Similar experiments were performed using a riboprobe directed against *xpnpep2*, but there was no staining in 3 dpf larvae (not shown).

Genes of interest *add3a* and *xpnpep1* are found on zebrafish chromosome 22 and are syntenic to their human counterparts, although they are not directly adjacent in zebrafish (*www.ensembl.org*, Zv9). These findings suggest that zebrafish *add3a* and *xpnpep1* are orthologous to the human genes. Nevertheless, we examined members of the *add3* and *xpnpep* families for liver expression during biliary development, because there are several possible orthologs for *add3* and *xpnpep1* in the fish. Both *add3a* (Fig. 1C) and *xpenpep1* (Fig. 1E) showed expression in the developing liver. Thus, we chose to inhibit *add3a* and *xpnpep1* to determine whether loss would lead to biliary defects and activation of pathways similar to those observed in BA.

### Knockdown of *add3a*, Not *xpnpep1*, Leads to Biliary Defects

**FIGURE 2:**
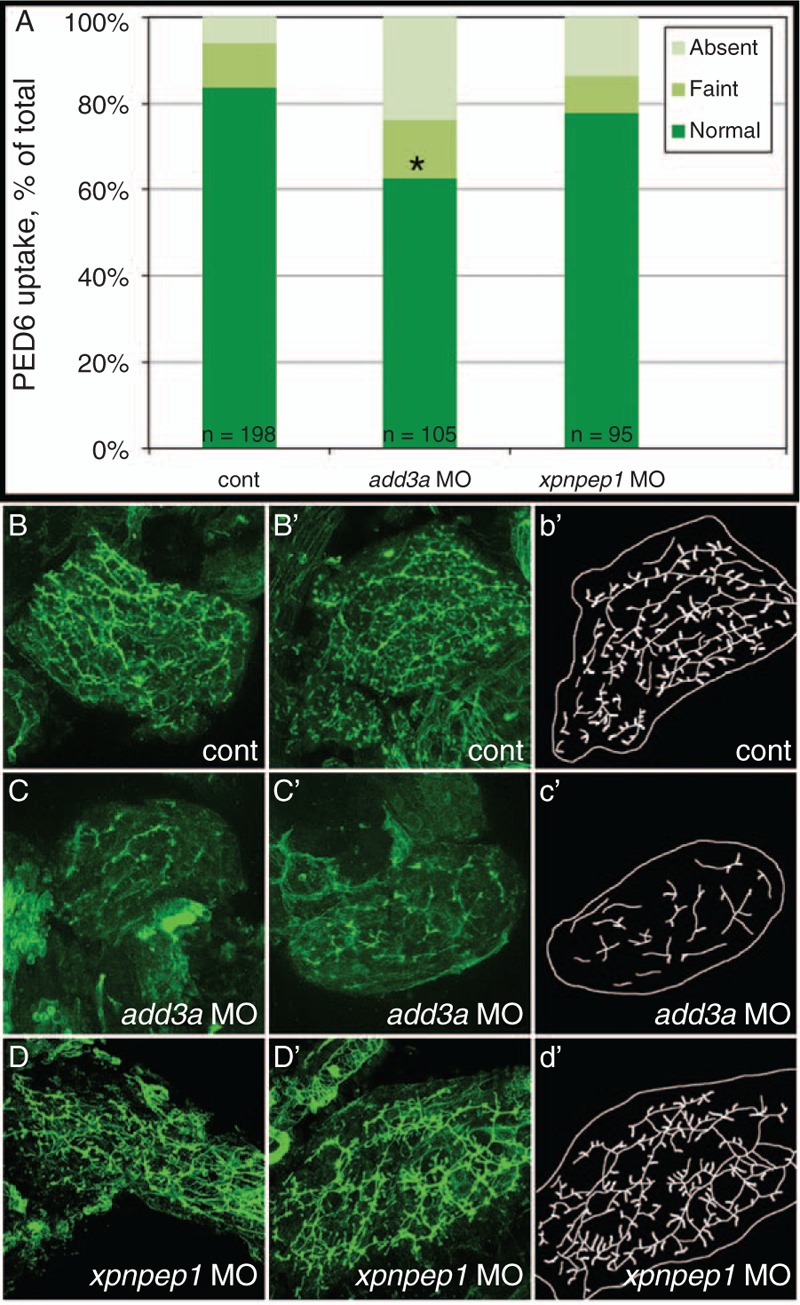
Decreased biliary function and developmental biliary abnormalities in *add3a* morpholino-injected 5 days post fertilization (dpf) larvae. Wild-type fertilized oocytes were injected with MO directed against *add3a* or *xpnpep1*, or control. At 5 dpf, larvae were incubated in the fluorescent lipid PED6 and assayed for gallbladder uptake, and then fixed and stained for cytokeratin to document biliary anatomy. A, Percentages of control (cont), *add3a* MO-injected and *xpnpep1* MO-injected larvae with normal, faint, or absent gallbladder visibility, reflecting PED6 uptake and thus biliary function. There is a significant decrease in biliary function in the *add3a* MO-injected larvae (^∗^*P* ≤ 0.0001 by chi-square analysis), whereas the difference between control and *xpnpep1* MO-injected larvae is not significant. B, Confocal projections of cytokeratin immunostainings of livers from 5 dpf larvae injected with control (cont, B), *add3a* MO (C), or *xpnpep1* MO (D). There are 2 examples of each condition (B, B’; C, C’; D, D’), and a schematic outline of each second example (b’, c’, d’). Note that the pattern of the intrahepatic ducts from the cont and *xpnpep1* MO-injected larvae appears similar, whereas the *add3a* MO-injected larvae have shorter and sparser ducts. Original magnification ×400. MO = morpholino antisense oligonucleotide.

To determine whether *add3a* and/or *xpnpep1* have a functional role in mediating biliary defects, we used MO knockdown to reduce expression of the mature mRNA and decrease protein expression (15). Although *add3a* morphants showed decreased biliary function as measured by PED6 uptake (Fig. 2A), *xpnpep1* knockdown had no significant effect on function (Fig. 2A). Cytokeratin staining showed obvious biliary abnormalities in the *add3a* morphants (Fig. 2C and C’), although *xpnpep1* knockdown had only a minor effect on biliary morphology (Fig. 2D and D’) when compared to controls (Fig. 2B and B’). In addition, MO-mediated knockdown of *add3a* phenocopies genetic deficiency (Supplemental Digital Content, Fig. 2), as *sa819* mutants demonstrate impaired biliary function (Supplemental Digital Content, Fig. 2B and C) and intrahepatic defects (Supplemental Digital Content, Fig. 2E and F), similar to morpholino-mediated knockdown. These results indicate *add3a* insufficiency, not *xpnpep1*, can result in biliary disease.

### Knockdown of *add3a* Leads to Hedgehog Activation

We have previously demonstrated that *GPC1*, which encodes *glypican-1*, is a BA susceptibility gene. MO-mediated knockdown of *gpc1* led to biliary defects similar to those shown here with *add3a* knockdown, and were associated with increased Hh activity (15). Hh activation is closely linked to liver fibrosis (21) and to BA (22), and thus we hypothesized that *add3a* deficits may also lead to increased Hh activity. To determine whether *add3a* was also affecting Hh activity, we examined transcriptional expression of Hh targets in *add3a* morphants.

**FIGURE 3:**
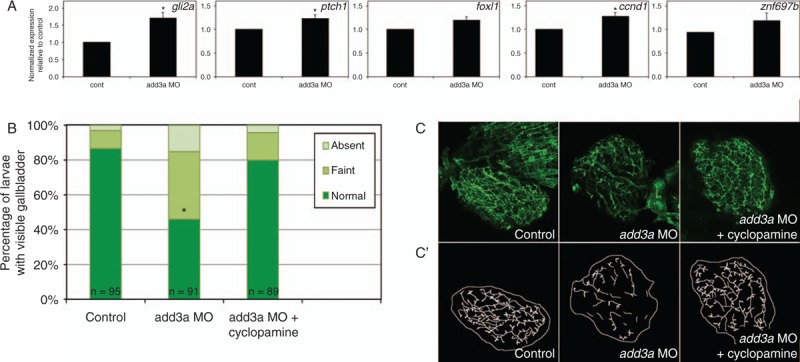
Studies of the Hedgehog (Hh) pathway in *add3a* morpholino-injected larvae. A, Quantitative RT-PCR of Hh pathway targets in control (cont) or *add3a* MO-injected larvae at 5 days post fertilization (dpf). Note that there is an increase in expression of all 5 genes shown (*gli2a*, *ptch1*, *foxl1*, *ccnd1*, *znf697b*), although only *gli2a*, *ptch1*, and *ccnd1* demonstrate a significant difference (^∗^*P* ≤ 0.05 by Student *t* test). B, C, Cyclopamine treatment rescues biliary defects in *add3a* morpholino-injected larvae. Wild-type fertilized oocytes were injected with MO directed against *add3a* or control. At 2 dpf, cyclopamine or vehicle was added, and at 5 dpf, larvae were incubated in the fluorescent lipid PED6 and assayed for gallbladder uptake, and then fixed and stained for cytokeratin to document biliary anatomy. B, Percentages of control (cont), and vehicle or cyclopamine-treated *add3a* MO-injected larvae with normal, faint, or absent gallbladder visibility, reflecting PED6 uptake and thus biliary function. There is a significant decrease in biliary function in the *add3a* MO-injected larvae (^∗^*P* ≤ 0.0001 by chi-square testing), which is significantly different from the cyclopamine treated larvae, with the same p-value. C, Confocal projections of cytokeratin immunostainings of livers from 5 dpf larvae injected with control, *add3a* MO, or *add3a* MO with cyclopamine. C’) Schematic tracings of the cytokeratin stainings shown in (C). Original magnification ×400. MO = morpholino antisense oligonucleotide.

Consistent with an increase in Hh pathway activity, *add3a* morphants showed increased expression of Hh target genes. Significant increases in expression were seen for *gli2a*, *ptch1*, and *ccnd1* (Fig. 3A). Interestingly, similar qPCR analysis of other genes important in biliary development and disease such as the Notch signaling pathway and Hnf6/Hnf1β did not demonstrate changes in expression (Supplemental Digital Content, Table 2). These results suggest *add3a* knockdown may specifically affect Hh pathway activity.

### Inhibition of the Hedgehog Pathway Protects Against Biliary Defects

Quantitative analyses indicated that Hh transcriptional activity was higher in *add3a* morphants, suggesting that the biliary defects in *add3a* morphants could be related to Hh overactivity. To determine whether Hh overexpression has a role in the biliary defects, we inhibited the Hh pathway using the Hh receptor antagonist, cyclopamine, in *add3a* morphants. Incubation of *add3a* morphants with cyclopamine resulted in normalization of biliary function as measured by PED6 uptake comparable to control animals (Fig. 3B), and a morphological rescue of the biliary defects (Fig. 3C). These results again support the hypothesis that biliary defects observed in *add3a* are caused by Hh pathway overexpression.

### The *add3a* and *gpc1* Genes Share Functional Characteristics in Mediating Biliary Development

**FIGURE 4:**
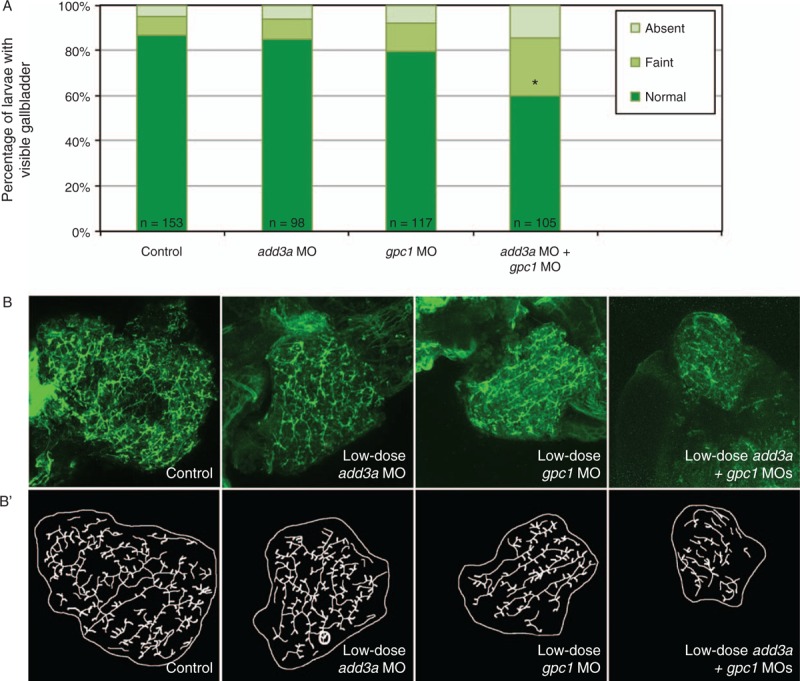
Epistatic effect of coinjection of *add3a* and *gpc1* morpholinos on biliary function and development. Wild-type fertilized oocytes were injected with MO directed against *add3a* or *gpc1* at lower amounts needed to see an effect, or control. At 5 days post fertilization (dpf), larvae were incubated in the fluorescent lipid PED6 and assayed for gallbladder uptake, and then fixed and stained for cytokeratin to document biliary anatomy. A, Percentages of control (cont), low-dose *add3a* MO-injected, low-dose *gpc1* MO-injected, or *add3a* and *gpc1* MO-injected larvae with normal, faint, or absent gallbladder visibility, reflecting PED6 uptake and thus biliary function. There is a significant decrease in biliary function only in the larvae injected with the lower dose of both MOs (^∗^*P* ≤ 0.0001 by chi-square analysis). B, Confocal projections of cytokeratin immunostainings of livers from 5 dpf larvae injected with control (cont), low-dose *add3a* MO, low-dose *gpc1* MO, or the combination of both MOs at the lower dose. B’, Schematics of the respective panels shown in (B). Note that the intrahepatic duct pattern is similar in the control and the larvae injected with *add3a* or *gpc1* MO alone, but that when the MOs are injected together the pattern is abnormal, with decreased duct density and complexity. Original magnification ×400. MO = morpholino antisense oligonucleotide.

We have previously used coinjection of MOs directed against targets in the same pathway to demonstrate an epistatic effect, supporting a combinatorial effect for the 2 genes (23). Because both *add3a* and *gpc1* knockdown lead to biliary defects and Hh activation, we hypothesized that *add3a* and *gpc1* function together to mediate biliary defects. Combined MO-mediated knockdown of *add3a* and *gpc1* led to decreased biliary function and to biliary defects at doses that did not lead to abnormalities when injected alone (Fig. 4). These results support that *add3a* and *gpc1* can work synergistically in the Hh pathway to mediate biliary defects.

## DISCUSSION

Our results demonstrate that *ADD3*, not *XPNPEP1*, is a putative candidate susceptibility gene for BA. Using zebrafish, we show that MO-mediated knockdown of *add3a* leads to impaired biliary function and developmental biliary anomalies. Zebrafish with mutations in *add3a* has similar biliary developmental defects. Knockdown of *add3a* leads to activation of Hh signaling, and the biliary defects are reversed by inhibition of Hh signaling. Zebrafish injected with morpholinos against *gpc1*, which was identified as a candidate susceptibility gene in a study of copy number variation in patients with BA (24), demonstrate similar features to the *add3a* knockdown and mutant embryos. Moreover, *add3a* and *gpc1* knockdown work additively in mediating biliary defects. Thus, like *GPC1*, *ADD3* appears to be a BA susceptibility gene that may function through modulating Hh signaling.

### Biliary Atresia Susceptibility Locus on Chromosome 10q24.2

In 2010, a genome wide association study performed in a Chinese population identified a BA susceptibility locus on chromosome 10q24.2 with a *P* value of 6.94 × 10^–9^(5). The most significant single nucleotide polymorphism was located between 2 genes, *XPNPEP1* and *ADD3.* This finding was later replicated in a Caucasian population, with the strongest signal in intron 1 of the *ADD3* gene (7). In that study, ADD3 protein was localized to intra- and extrahepatic bile ducts, and *ADD3* gene expression was increased overall in BA liver as compared with normal control liver. In light of the specific localization of ADD3 to bile ducts, it is possible that the increased *ADD3* expression in whole BA liver may be explained by bile ductular proliferation in these samples. A correlation between single nucleotide polymorphism genotype and *ADD3* expression level was not identified in a small number of samples in that study (7). In 2013, Cheng et al (25) reported an exhaustive analysis of the 10q24.2 chromosomal region, in which they demonstrated a correlation between the risk allele and reduced expression of *ADD3* specifically in BA liver. No such correlation was found for *XPNPEP1*. They also noted higher levels of *ADD3* expression in liver obtained at the time of transplantation as compared with samples collected at time of Kasai, indicating that stage of disease may play a role. Taken together, the data suggest that the risk allele identified in the GWAS may result in a modest decrease in *ADD3* gene expression in BA liver, but further studies will be required to determine the downstream effects of altered *ADD3* expression at specific stages of development and disease progression.

### Adducin Abnormalities and Biliary Defects

*ADD3* encodes gamma-adducin, which forms tetramers at actin-spectrin junctions and interacts with calmodulin and gelsolin to tether the cytoskeleton to the plasma membrane. Adducin is also important in cell movement, in the outgrowth of neurites, in the stabilization of epithelial junctions, and in the regulation of cell adhesion and cell polarity (26). In addition to other interactions, the adducin tetramer acts as a substrate for Rho-dependent kinases. Rho kinases are key regulators of cell contraction, migration, and cell polarity (27), and the phosphorylation of adducin by Rho-kinase promotes actin binding (28).

Normal epithelial polarity is essential for normal liver and biliary development (29,30). Hepatocyte polarity is lost in cholestatic liver injury, leading to further dysregulation of bile transport pathways (29,31). Interestingly, disruption of the actin cytoskeleton has been shown to induce cholestasis, because rats treated with phalloidin have decreased bile flow. There is altered hepatocyte polarity in these animals, as evidenced by disordered trafficking of canalicular proteins to the basolateral domain and vice versa (32,33). We have previously shown that disruption of planar cell polarity can lead to biliary defects, and these defects are in fact synergistic with inhibition of the actin cytoskeleton (19). Genetic disruption of adducin function could impair hepatocyte or cholangiocyte polarity and/or movement, which would then have an effect on bile duct development.

As noted above, we detected modest activation of Hh pathway targets in our *add3a* morphants, similar to what was observed in BA and in other cholestatic disorders (22), and inhibition of the Hh pathway rescued the *add3a* phenotype in zebrafish. Interestingly, actin-mediated cellular changes affect Hh signaling (34), and Hh signaling is also necessary for the stabilization of the actin cytoskeleton and tight junctions (35). Thus, disruption of *add3* could lead to poor epithelial connections, disorganized actin cytoskeleton, and a loss of cell polarity that results in activation of Hh, which then leads to inflammation, fibrogenesis, and perhaps developmental biliary defects that perpetuate a cycle of Hh activation.

### Assimilating Biliary Atresia Genetic Risk

The etiology of BA is most likely multifactorial, with environmental triggers such as toxins and infections combined with inflammatory and genetic risk factors (36). For example, the risk haplotype associated with *ADD3* on chromosome 10q24.2 is relatively common, occurring in 49% of Chinese individuals with BA and 32% of controls (25). It is likely that this risk allele imparts a susceptibility to BA in combination with another insult, such as an infection or toxin. There may in fact be several possible causes of BA that manifest in different patients, with different triggers and risk factors resulting in a similar phenotype. Although BA is not strictly a genetic disease, there has been prior evidence suggesting genetic involvement. The incidence is higher in the Asian population compared to Caucasians with incidence of 1 in 12,000 to 18,000 in Western Europe versus 1.04 to 3.7 per 10,000 in Japan and Taiwan (1,37,38). We have previously demonstrated a functional role for *GPC1* as a susceptibility gene for BA, and in that report we summarized previous studies linking genes such as *JAG1*, *CFC1*, *ZEB2*, and others (15). Continuing broad-based genetic studies attempt to understand the contribution of these and other similar genes. Results from these studies may allow us to identify patients at risk for BA.

Perhaps more important than the individual genes uncovered in these studies are the pathways and cellular processes affected by the genes. Abnormally activated Hh signaling appears to be important in BA and in animal models, and thus continued examination of mechanisms that lead to activation of Hh may lead to insights regarding pathogenesis. The accumulated genetic studies also suggest an importance of cilia and polarity pathways, which are connected to Hh activity as well. Supporting this importance of polarity pathways, mice haploinsufficient in *Sox17* demonstrate abnormally polarized cholangiocytes, leading to shedding of the cholangiocytes into the bile duct lumen and resulting in obstruction (39). Interestingly, a recent study has identified a novel plant isoflavonoid toxin that has been implicated in outbreaks of BA in Australian livestock (40). Treatment with this toxin results in biliary defects in zebrafish larvae, and in a neonatal cholangiocyte spheroid model the toxin causes loss of cell polarity and obstruction of the lumen. Taken together, these findings suggest a common pathway for genetic and toxin-mediated etiologies of BA, in which intrinsic defects in cholangiocyte polarity lead to a more distal duct obstruction, which then would lead to BA.

Our results demonstrate that inhibition of *add3a*, a zebrafish ortholog of human *ADD3*, first identified by GWAS as a candidate BA susceptibility gene, causes abnormalities in biliary structure and function in zebrafish. Furthermore, blockade of the Hh pathway rescues the phenotype, indicating that *ADD3* may be acting as part of the Hh pathway. The known functions of *ADD3* in cell adhesion and polarity could explain the mechanism of its involvement in BA pathogenesis. Thus, the genetic studies on BA appear to be advancing us toward uncovering pathogenic mechanisms leading to disease.

## Supplementary Material

Supplemental Digital Content
