# Supplementary material for: Loss of a Candidate Biliary Atresia Susceptibility Gene, add3a, Causes Biliary Developmental Defects in Zebrafish
Source: J Pediatr Gastroenterol Nutr. 2016 Oct 24;63(5):524–30. doi: 10.1097/MPG.0000000000001375 (PMC5074882; doi:10.1097/MPG.0000000000001375)
Supplement: Supplemental Digital Content [file jpga-63-524-s001.pdf]

Supplementary Table 1

**Primer sequences for *in situ* hybridization**	
DR xpnpep1-F1ish	CCAAAGATCACGGTGGAGCT
DR xpnpep1-R1ish	GAATTCATTAACCCTCACTAAAGGGACGAATCGCAGGGTCCGATAA
DR xpnpep2-F1ish	CAAGGCTGTTTTGTGGACCG
DR xpnpep2-R1ish	GAATTCATTAACCCTCACTAAAGGGAAGGTACTGTCCTCCGGAGTC
DR add1-F1ish	GTCCGGCTGAACTCTGAACA
DR add1-R1ish	GAATTCATTAACCCTCACTAAAGGGAGAACGGTCAACAACAGCACC
DRadd2-F1ish	AGGCTTTGTTGGTGGGTGAA
DRadd2-R1ish	GAATTCATTAACCCTCACTAAAGGGATATGGGGGAGGTAGTTGGGG
DRadd3a-F1ish	CGCAGACTTCTTCATGTCCA
DRadd3a-R1ish	GAATTCATTAACCCTCACTAAAGGGATTCACTGGGGTTTGTGTTCA
DRadd3b-F1ish	ATCTGTGTGGCATGGAGTGG
DRadd3b-R1ish	GAATTCATTAACCCTCACTAAAGGGAGTCTGCCAGCAGTTGAGACT
**MO sequences**	
xpnpep1-5'	ACATTGTGTCTGTTGGGAAACAAAC
xpnpep1-IE3	TCACTCTACAGAGAAGGATACACAC
add3a-5'	TCCTGTCTCGGCTCGCCACTCATCT
add3a-IE3/6	AACGCCTGAAAGAGAATGTGAGTT
Standard Control	CCTCTTACCTCAGTTACAATTTATA
**Genotyping primer sequences for sa819**	
add3a_sa819F	CCAGGAATTCAAATTGGTAGGA
add3a_sa819R	CAAGGCTCAGTTTGATGGTG

Supplementary Table 1. **Sequences of *in situ* hybridization primers, morpholinos (MOs) and genotyping primers**. Note that the reverse primers for *in situ* hybridization have a T3 sequence that allows synthesis of riboprobe directly from the PCR product.

Supplemental Table 2

	**Expression in *add3a* MO-injected relative to control**	**Family**
vhnf1	1.00±0.08	HNF6 target
her6	1.02±0.04	Jagged/Notch target
ctgf	1.04±0.28	TGFβ/fibrosis target
irf1b	1.56±0.52	Interferon-gamma target

Supplemental Table 2. Genes with no significant difference in *add3a*-injected larvae. Quantitative PCR studies of 5 dpf larvae (torsos) with no difference in expression. Shown are primer pairs for *vhnf1* (*hnf1b, tcf2*), an HNF6 target, *her6* (Notch target), *ctgf* (a TGFβ target that is also associated with fibrogenesis), and *irf1b* (an interferon-gamma target). Differences are not significant between the *add3a* MO-injected condition and control (Student's t-test).

Supplemental Figure 1

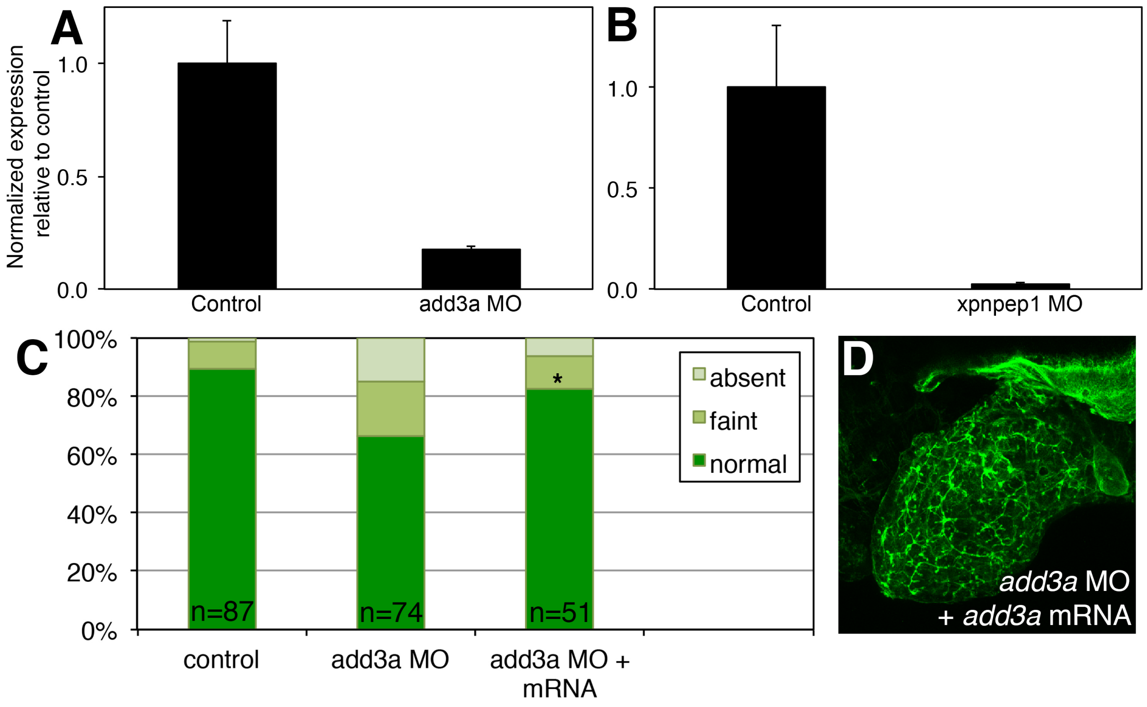

Documentation of morpholino effect. (A-B) Quantitative PCR expression of product with 3' primer in deleted exon, showing markedly decreased expression of *add3a* (A) and *xpnpep1* (B). (C) Gallbladder PED-6 uptake from control, *add3a* MO-injected, and *add3a* MO and mRNA-injected larvae, showing statistically significant rescue of the *add3a* MO phenotype by *add3a* mRNA injection. (D) Confocal projection of liver cytokeratin staining of mRNA-rescued 5 dpf larva, showing an essentially normal phenotype. *$p \leq 0.0001$, chi square test, MO- + mRNA-injected vs. MO-injected.

Supplementary Figure 2

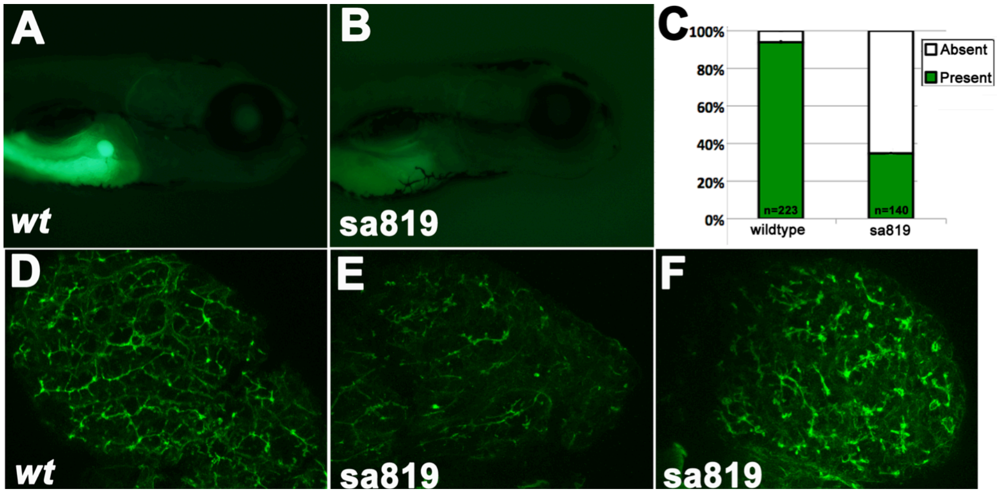

***add3a* deficiency causes defects in biliary function and morphology.** Right lateral views of 5dpf larvae after PED-6 ingestion showing the presence (A) or absence (B) of a gallbladder. (C) Percentages of wildtype siblings and sa819 larvae (*$p \leq 0.0001$ by chi-square analysis) with present or absent gallbladder visibility, reflecting PED6 uptake and thus biliary function. (D-F) Confocal projections of cytokeratin immunostainings of livers from (D) wild-type or (E,F) sa819 mutants at 5 dpf. Note that the pattern of the intrahepatic ducts from sa819 mutant larvae appears similar to *add3a* MO-injected larvae from the previous figure. Original magnification 400x.
